# Impact of Age and Race on Outcomes of a Program to Prevent Excess Weight Gain and Disordered Eating in Adolescent Girls

**DOI:** 10.3390/nu9090947

**Published:** 2017-08-28

**Authors:** Natasha L. Burke, Lauren B. Shomaker, Sheila Brady, James C. Reynolds, Jami F. Young, Denise E. Wilfley, Tracy Sbrocco, Mark Stephens, Cara H. Olsen, Jack A. Yanovski, Marian Tanofsky-Kraff

**Affiliations:** 1 Department of Medical and Clinical Psychology, Uniformed Services University of the Health Sciences (USUHS), 4301 Jones Bridge Road, Bethesda, MD 20814, USA; Natasha.Burke.ctr@usuhs.edu (N.L.B.); Tracy.Sbrocco@usuhs.edu (T.S.); 2 Section on Growth and Obesity, *Eunice Kennedy Shriver* National Institute of Child Health and Human Development (NICHD), National Institutes of Health (NIH), US Department of Health and Human Services (DHHS), 10 Center Drive, Bethesda, MD 20892, USA; BradyS@mail.nih.gov (S.B.); JY15i@nih.gov (J.A.Y.); 3 Department of Human Development and Family Studies, Colorado State University, 303 Behavioral Sciences Building, 1570 Campus Delivery, Fort Collins, CO 80523, USA; Lauren.Shomaker@colostate.edu; 4 Radiology and Imaging Sciences Department, Warren Grant Magnuson Clinical Center, National Institutes of Health (NIH), US Department of Health and Human Services (DHHS), 10 Center Drive, Bethesda, MD 20892, USA; JReynolds@cc.nih.gov; 5 Department of Child and Adolescent Psychiatry and Behavioral Sciences, Children’s Hospital of Philadelphia, 3401 Civic Center Blvd., Philadelphia, PA 19104, USA; YoungJF@email.chop.edu; 6 Department of Psychiatry, Washington University School of Medicine, 660 South Euclid, Campus Box 8134, St. Louis, MO 63110, USA; WilfleyD@psychiatry.wustl.edu; 7 Department of Family and Community Medicine, Pennsylvania State University, 1850 E. Park Avenue, Suite 207, State College, PA 16803, USA; MStephens3@pennstatehealth.psu.edu; 8 Department of Preventative Medicine & Biometrics, Uniformed Services University of the Health Sciences (USUHS), 4301 Jones Bridge Road, Bethesda, MD 20814, USA; Cara.Olsen@usuhs.edu

**Keywords:** loss-of-control eating, obesity, BMI, adiposity, adolescent, age, race, interpersonal psychotherapy

## Abstract

Interpersonal psychotherapy (IPT) prevents weight gain and reduces loss-of-control (LOC)-eating in adults. However, IPT was not superior to health-education (HE) for preventing excess weight gain and reducing LOC-eating over 1-year in adolescent girls at risk for excess weight gain and eating disorders. Limited data suggest that older and non-White youth may be especially responsive to IPT. In secondary analyses, we examined if age or race moderated weight and LOC-eating outcomes. The 113 participants (12–17 years; 56.6% White) from the original trial were re-contacted 3 years later for assessment. At baseline and follow-up visits through 3 years, we assessed BMI, adiposity by dual energy X-ray absorptiometry, and LOC-eating presence. In linear mixed models, baseline age moderated 3-year BMI outcome; older girls in IPT had the lowest 3-year BMI gain compared to younger girls in IPT and all girls in HE, *p* = 0.04. A similar pattern was observed for adiposity. Race moderated 3-year LOC-eating; non-White girls in IPT were most likely to abstain from LOC-eating at 3 years compared to all other girls, *p* = 0.04. This hypothesis-generating analysis suggests future studies should determine if IPT is especially efficacious at reducing LOC-eating in older, non-White adolescents.

## 1. Introduction

Excess body weight continues to be a source of health problems in youth [1]. Over 34% of adolescents in the United States currently have overweight or obesity (body mass index (BMI) ≥ 85th percentile; [2]). The prevalence of severe obesity (BMI ≥ 120% of the 95th percentile) during adolescence appears to be climbing particularly rapidly [3], rendering this demographic in urgent need for intervention. As evidenced in previous weight management trials [4,5,6], age may be a potential moderator of obesity treatment effects. In family-based behavioral treatment studies, younger children achieve better weight outcomes than older children [7]. Indeed, although family-based behavioral treatment is considered a first line intervention among middle childhood youths, its efficacy for adolescents is less robust [8]. Yet, adolescence is a key developmental stage as it relates to the development of disordered eating behaviors, including loss-of-control (LOC)-eating, that may lead to excess weight gain [9]. In addition, it is a crucial time for changes in the types, number, and nature of social relationships [10] that can affect overall health, functioning, and mood [11].

Race also requires important consideration in obesity intervention efforts. Adolescents of certain ethnic and racial minority groups are at disproportionately heightened risk for developing overweight and obesity. Data indicate that 38.1% of Hispanic and 39.8% of non-Hispanic Black youth ages 12–19 meet criteria for overweight or obesity [2]. Unfortunately, obesity intervention efforts broadly focused on energy balance have largely been ineffective at reducing current prevalence rates in racial and ethnic minority groups [12,13,14,15]. Both age and race/ethnicity likely play important roles in intervention approach, and factors related to both of these demographic dimensions should be considered in excess weight gain prevention efforts. In particular, innovative approaches to address older adolescents and those of non-White racial and ethnic minority groups is crucial, given the difficulties in identifying effective interventions in these at-risk youth.

Loss-of-control (LOC)-eating is a robust predictor of excess weight and fat gain over time [16,17,18]. A hallmark of binge-eating disorder, LOC-eating is characterized by the subjective feeling that one cannot stop eating, regardless of the amount of food consumed [19]. Notably, LOC-eating appears to manifest in middle childhood and shows comparable prevalence among adolescents from diverse racial and ethnic groups [20,21]. It is also common among youth who are overweight [20], with lifetime prevalence rates up to 45% in adolescents seeking treatment for obesity [22]. In addition to prospective links to weight and fat gain [16,17,18], LOC-eating predicts a host of detrimental psychological [18,20,23] and physical [24,25] health problems. Therefore, we have proposed reducing LOC-eating as a highly targeted approach to preventing excess weight gain in youth who experience this disordered eating pattern [26]. Given data showing that interpersonal psychotherapy (IPT) has been effective at reducing weight gain and LOC-eating in adults with obesity and binge-eating disorder, we conducted a randomized controlled trial aimed at reducing excess weight gain and LOC-eating episodes in adolescents at high risk for both conditions [27]. In contrast to expectations, we found that IPT was not superior to a health-education (HE) control condition for preventing excess weight gain at 1- or 3-year follow-up in 113 adolescent (12–17 years) girls with LOC-eating and high BMI (kg/m^2^; [27,28]). With the absence of main effects, we sought to examine the heterogeneity in age and race/ethnicity in this cohort as possible moderators of treatment outcomes. Given the broad age range and racial/ethnic diversity of our sample, and the role of these factors in weight management interventions in past studies [4,5,6], it is possible that demographics play an important moderating role in weight and eating outcomes.

We therefore conducted secondary, hypothesis-generating analyses to examine the impact of baseline age and race/ethnicity on 3-year weight and eating outcomes from our original trial. Considering IPT’s focus on interpersonal stressors key to adolescence [26,29], we hypothesized that older adolescents randomized to IPT would respond better than younger adolescents randomized to IPT or HE and older adolescents randomized to HE. This expectation is consistent with data from a school-based effectiveness study for depression, which found that IPT was more effective than treatment-as-usual for older versus younger adolescents [30]. Moreover, given data suggesting that racial/ethnic minorities may be more responsive to IPT than other modalities [31], we hypothesized that treatment effects for racial and ethnic minority girls randomized to IPT would supersede those of girls from any other group.

## 2. Method

### 2.1. Participants

From the original study, 113 adolescent girls, 12–17 years (Mean = 14.50, Standard Deviation = 1.65), participated in a randomized controlled clinical trial (ClinicalTrials.gov ID#: NCT00680979) to prevent excess weight gain and eating disorders [27]. Of these, 68 girls agreed to participate in an additional 3-year follow-up time-point that was not part of the originally-funded trial [28]. As previously described [27], inclusionary criteria involved being between the 75th and 97th BMI percentiles and endorsing at least one episode of LOC-eating in the past month. Exclusionary criteria included major medical or psychiatric diagnoses (other than binge-eating disorder), medication that affected weight or appetite, and concurrent participation in psychotherapy or a structured weight loss program. Parental consent and adolescent assent (or consent if girls were 18 years or older at 3-year follow-up) were obtained prior to study procedures. The study was approved by the institutional review boards of the Uniformed Services University and *Eunice Kennedy Shriver* National Institute of Child Health and Human Development.

### 2.2. Procedures

At baseline, demographic variables (e.g., age and race/ethnicity) were assessed by parent report. Socioeconomic status was assessed with the Hollingshead index [32]. At baseline, immediately post-group, 6-month, 1-year, and 3-year follow-ups, participants were assessed at the National Institutes of Health Clinical Research Center following an overnight fast initiated the night before at 10 p.m.

*BMI and Adiposity*. Weight was measured to the nearest tenth of a kilogram with a calibrated digital scale (Scale-Tronix, Wheaton, IL, USA). Height was measured in triplicate to the nearest millimeter with a calibrated electronic stadiometer (Holtain Ltd., Crymych, Wales, UK); the average of the three heights was used in the analyses. Weight and height were measured using the same assessment methods over time. BMI was calculated from measured height and weight (kg/m^2^). Fat mass (kg) was measured by dual energy X-ray absorptiometry (DXA) using a Hologic QDR-4500A or Discovery instrument (Bedford, MA, USA). There were no discrepancies between instruments [27]. BMI was assessed at baseline, immediately post-group, and at 6-month, 1-year, and 3-year post-intervention visits. Fat mass was determined at all time points, except post-group.

*LOC-Eating*. The presence of LOC-eating episodes over the past month was assessed at each time point by the overeating section of the Eating Disorder Examination, Version 14 OD/C.2 (EDE; [33]). The Eating Disorder Examination is a semi-structured diagnostic clinical interview that measures disordered eating behaviors and has been shown to be reliable and valid for the assessment of LOC-eating in adolescent samples of broad age ranges and racially and ethnically diverse samples [22,34,35].

### 2.3. Randomization

Participants were randomized to an adapted version of IPT or a HE comparison group, as previously described [27]. The IPT group was adapted from two manualized IPT programs, IPT-Adolescent Skills Training for the prevention of depression [36] and group IPT for BED [37]. In IPT, participants were taught to connect their interpersonal relationship difficulties with their mood and eating. Girls learned communication skills to address challenging interpersonal problems with the goal of improving mood and reducing negative affect-induced LOC-eating that promotes excessive weight gain [27]. The HE group was adapted from Duke University’s “Hey-Durham” HE program for high school students [38]. It includes a range of HE topics covering mental, social, and physical functioning, including modules on nutrition and body image [38]. The Hey-Durham curriculum was adjusted to time-match the session length and frequency of IPT, which included a 90-min pre-group individual meeting with each girl, followed by 12 consecutive, weekly 90-min group sessions [27]. Both IPT and HE groups were led by a trained PhD-level clinical psychologist and a doctoral student in clinical psychology [27]. Program delivery and treatment fidelity were independently rated, and adherence to treatment condition was excellent [27].

### 2.4. Statistical Analyses

All analyses were conducted with SPSS Version 24 (IBM Corporation, Chicago, IL, USA). The primary outcomes were BMI, adiposity, and LOC-eating status. Age and race were investigated separately as moderators for each outcome variable. Due to the relatively small number of girls who were racial/ethnic minorities, race was operationalized as non-Hispanic White vs. all other racial/ethnic groups. Binary logistic regression was used to evaluate the interactions of group by age or race on LOC-eating status at 3 years, adjusting for post-intervention LOC-eating status (i.e., presence or absence of LOC-eating) and socioeconomic status (SES; [32]). Linear mixed models with restricted maximum likelihood estimation for handling missing data were used to evaluate the interactions of group by time with age or race for BMI and adiposity at 3 years. Time was calculated as number of weeks from baseline to account for variability in timing from post-assessments (approximately 12 weeks, 6 months, 1 year, and 3 years post-treatment). Models for adiposity covaried for height, and all models adjusted for SES. Linear mixed models included two- and three-way interactions with group and time as model-fixed effects.

## 3. Results

One-hundred and sixty-six participants were assessed at baseline, and 113 eligible youth were enrolled and randomized [27]. Please refer to Tanofsky-Kraff et al., 2014, for a full CONSORT flow diagram [27]. Descriptive statistics for participants at baseline and 3-year follow-up are presented in Table 1. At baseline, girls randomized to IPT were somewhat younger and reported fewer LOC-eating episodes compared to girls in HE, but were otherwise similar [27]. Of the 113 participants at baseline, 60% completed the 3-year follow-up with no significant differences in follow-up for those randomized to IPT (*n* = 36) versus HE (*n* = 32) [28]. Demographics including age, race, BMI, and adiposity were not significant predictors of attrition [28]. As previously reported [28], there was no main difference between IPT and HE in LOC-eating presence, BMI, adiposity, anxiety symptoms, or depressive symptoms at 3-years.

### 3.1. Interactions between Group Assignment and Age/Race for 3 Years BMI and Adiposity

**Figure 1:**
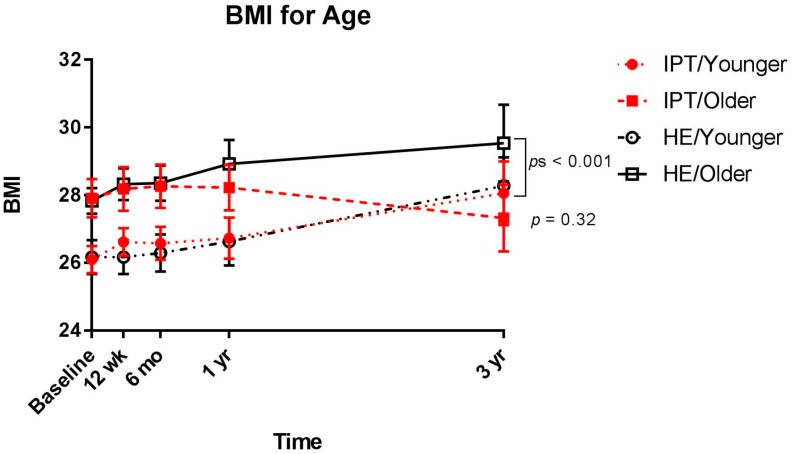
Change in body mass index (BMI) over a 3-year follow-up in adolescent girls randomized to interpersonal psychotherapy (IPT) or health education (HE) by median split age (younger: 12.02–14.05 years vs. older: 14.18–17.83 years).

Baseline age was a significant moderator of the effect of group on BMI over time, *F*(1, 372) = 3.94, *p* = 0.048. Post hoc analyses based on median age split revealed that, among younger participants (ages 12.02–14.05 years), those assigned to HE or IPT had significant gains in BMI over the 3-year period (*p*s < 0.001). Among older participants (ages 14.18–17.83 years), there was a group effect, such that those in IPT stabilized BMI (*p* = 0.32), whereas older adolescents in HE had significant BMI gain (*p* < 0.001; Figure 1). Similar to the effect on BMI, there was a trend for age as a moderator on the effect of group on change in adiposity, *F*(1, 266) = 3.60, *p* = 0.059. Though not statistically significant, the trend was for older girls in IPT to stabilize in fat gain, whereas older girls in IPT and younger and older girls in HE gained in adiposity over 3 years. In contrast to age, race was not a significant moderator of the effect of group on BMI or adiposity over time, *F*(1, 376) = 0.01, *p* = 0.92 and *F*(1, 271) = 1.12, *p* = 0.29, respectively.

### 3.2. Moderating Effects of Age and Race on LOC-Eating Status at 3 Years

A test of the model against a constant-only model was not statistically significant (χ^2^ = 9.01, *p* = 0.11 with *df* = 5), meaning that there was no moderating impact of age on LOC-eating presence at 3 years. Group, age, the age by group interaction, and SES were not significant predictors in the model. Immediate post-intervention LOC-eating status was the only significant predictor, such that adolescents who endorsed LOC-eating directly after the intervention were more likely to continue to have LOC-eating at 3 years (Unstandardized coefficient = 1.61, Standard Error = 0.49, *p* = 0.001).

Race moderated the group effect on LOC-eating. Non-White girls in IPT were more likely to abstain from LOC-eating at 3 years compared to all other girls, *p* = 0.04 (Table 2). For race, a test of the model against a constant-only model was statistically significant (χ^2^ = 18.35, *p* = 0.03 with *df* = 5), indicating the predictors as a whole distinguished between those with and without LOC-eating at 3 years. Group, race, and SES were not significant individual predictors in the model. The odds ratio of the group effect on LOC-eating was 0.98 [0.23, 4.28] for White girls and 8.45 [0.64, 112.26] for non-White girls. However, post-intervention LOC-eating status and the race by group interaction were both significant, unique predictors.

### 3.3. Exploratory Follow-Up Analyses of Race Dichotomized as Black versus White

Considering the majority of non-White youth were Black, we conducted exploratory analyses dichotomizing race into Black and White groups. Similar to data for the total sample, race (Black vs. White) was not a significant moderator of the effect of group on BMI or adiposity over time, *p*s = 0.89 and 0.33, respectively. In contrast to data for the whole sample, race did not moderate the group effect on LOC-eating. However, the interaction coefficient was in the same direction, indicating the pattern of the result was similar.

## 4. Discussion

In this secondary analysis of results obtained 3 years after an intervention aimed at reducing excess weight gain and the presence of LOC-eating, we found that age moderated weight by intervention group outcomes and race moderated LOC-eating by intervention group outcomes. Specifically, older girls in IPT had the lowest 3-year BMI gain compared to younger girls in both conditions and older girls in HE. Further, non-White girls in IPT were more likely to abstain from LOC-eating at 3 years compared to all other girls. Accordingly, baseline demographics may be relevant for understanding IPT’s impact on weight and disordered eating.

Older girls randomized to IPT experienced the best BMI and adiposity outcomes. Most weight management data involve younger children, as opposed to adolescents [8]. Also, at least one study of youth with obesity found that children were more responsive to lifestyle approaches than adolescents [39]. Family-based behavioral interventions generally focus on parents and families working to make healthier food and activity decisions. In contrast, group IPT does not include a parent component and does not explicitly target energy balance-related behavioral goals. Instead, IPT addresses interpersonal difficulties and resulting negative mood states that prompt unhealthy eating behaviors [26]. Through adolescence, interpersonal relationships broaden beyond the realm of family to include a greater influence of peers and the key developmental task of establishing intimacy with individuals outside of one’s parents [10,11]. In addition, social concerns and mood fluctuations increase significantly during puberty, particularly among girls [11]. IPT addresses key areas of interpersonal stress that occur in the developmental transition from childhood to adolescence including role transitions, role conflicts, and interpersonal deficits [26,29]. Therefore, IPT may be particularly beneficial for older versus younger adolescents’ BMI outcomes, by addressing social problems that contribute to emotions and behaviors that promote a positive energy balance. The findings from the current study also mirror results from a depression randomized controlled trial with adolescents where older girls, who also had the most severe levels of depressive symptomology, evidenced better outcomes when randomized to IPT versus treatment-as-usual [30].

In contrast to our hypotheses, race did not significantly moderate group by time effects for BMI or adiposity. The categorization of participants into a dichotomous race variable may have obscured findings by introducing greater variance to the non-White group, which perforce included individuals from various racial and ethnic backgrounds. Due to sample size constraints in the current study, ethnic minority groups were categorized together. In addition, the etiology of excess weight gain is complex and multifaceted [40]. It is possible that investigation of additional factors, such as environmental and familial characteristics, may need to be considered to evidence moderating effects of race on weight outcomes over time.

Non-White girls were significantly less likely to report LOC-eating at 3 years if they were assigned to IPT. This finding is consistent with our results at 1-year follow-up such that non-White girls reported fewer LOC-eating episodes if they had received IPT [27]. Results from studies investigating the effectiveness of IPT for disordered eating in adults indicate that IPT may be more effective for racial and ethnic minorities than other therapy modalities, such as cognitive-behavioral therapy [31]. There are a number of possible explanations for this pattern. Certain racial and ethnic minority youth may be at increased risk for stressful life events [41,42], potentially due to racism, discrimination, micro-aggressions, and acculturative stress [43,44,45]. The theoretical model underlying IPT may be particularly appropriate for identifying the precipitating and maintaining factors of stressful life events that induce negative moods and trigger LOC-eating [41]. One possibility to explain why racial/ethnic minority youth in IPT were significantly less likely to endorse LOC-eating at 3 years might be that IPT can address culturally specific stressful life events that spur negative affect and disordered eating. Nevertheless, since race did not moderate IPT’s effects on change in body weight, it is also possible that culturally, normative (i.e., non-LOC-eating) meals might still promote excess body weight for certain ethnic minority girls [34]. Thus, while IPT may reduce disordered eating among these youths, behavioral modification education also may be required to reduce excess weight gain. For non-Hispanic White girls, intervention group did not moderate outcomes over time. This result is consistent with data at 1-year follow-up [27]. However, it may also speak to the increasing trajectory of disordered eating behaviors in non-Hispanic White girls over time [46]. Future studies might consider that to influence LOC-eating behaviors in this group over time, booster sessions post-intervention may be warranted.

In contrast to our hypotheses, age did not moderate LOC-eating status by treatment group at 3 years. Data show that for about 50% of preadolescent youths, LOC-eating remits after 2 to 5 years [23,47]. It is possible that, in spite of intervention group, LOC-eating remitted over time for a portion of the participants regardless of age. Yet, for those for whom LOC-eating does not remit, successful intervention is still needed.

Strengths of this investigation include the relatively long-term outcome of 3 years following a randomized controlled trial. In addition, use of linear mixed models for weight outcomes accounted for correlated data due to repeated measurements and missing observations across time periods [48]. Assessment of LOC-eating by clinician interview and objective measures of height, weight, and adiposity are also study strengths. Limitations include the dichotomization of race, which may obscure findings for racial and ethnic minority youth. Furthermore, the 3-year follow-up was not part of the initial study design. Still, the 60% retention rate was reasonable considering the 3-year follow-up was unplanned and there did not appear to be systematic differences in missingness [28]. Due to sample characteristics, findings may not be generalizable to girls without LOC-eating or to boys. Also, due to the secondary nature of analyses, hypothesis-driven replication is required.

The current study supports the need for further research into the impact of baseline age and racial demographics as moderators of weight and eating outcomes for IPT. Indeed, given that older adolescent girls have traditionally been excluded from, or had poor outcomes in, prior weight management trials, these data provide a promising avenue for future investigation. Our findings may be particularly important given the persistence of obesity into adulthood for adolescents with excessive weight [49]. Furthermore, our results bolster the adult literature indicating that IPT is particularly effective for reducing disordered eating in racial and ethnic minorities. Considering the relatively high rates of sub-threshold binge eating in adulthood for some racial/ethnic minority groups [50] and the known associations with negative psychological and physical health outcomes [20,25,51], it is encouraging that these outcomes may be abated during adolescence using IPT delivered in a group therapy format.

## Figures and Tables

**Table 1:** Descriptive statistics for participants at baseline and 3-year follow-up.

	IPT Group		HE Group	
	Baseline (*n* = 55)	3-Year (*n* = 36)	Baseline (*n* = 58)	3-Year (*n* = 32)
Age, years †	14.18 (1.52)	17.22 (1.59)	14.80 (1.73)	17.93 (1.80)
Race/ethnicity, %				
White	52.7	53.1	60.4	58.3
Black	25.4	25.0	22.4	19.4
More than one race	7.3	9.4	6.9	8.3
Asian	5.5	3.1	0	0
American Indian	0	0	1.7	2.8
Hispanic	9.1	9.4	8.6	11.1
BMI, kg/m^2^ †	26.86 (2.61)	27.78 (3.90)	27.08 (2.43)	28.89 (4.24)
Adiposity, kg †	26.38 (5.81)	27.74 (8.02)	26.40 (6.00)	28.68 (8.13)
Socioeconomic Status †	2.28 (0.94)	--	2.28 (0.94)	--
LOC-eating status, % present	100	60	100	40

Note: IPT = Interpersonal psychotherapy group; HE = Health education group; BMI = Body mass index, LOC = Loss-of-control-eating status past 3 months; † Mean ± Standard Deviation.

**Table 2:** Logistic regression for loss-of-control-eating status at 3 years.

	*B* (*SE*)	Odds Ratio	95% CI for Odds Ratio		*p* Value
			Lower	Upper	
Intercept	−0.66 (1.02)	0.52	--	--	0.52
SES	−0.53 (0.37)	0.59	0.29	1.22	0.15
Post-intervention LOC-eating status	1.70 (0.75)	5.46	1.26	23.69	0.02
Group	0.01 (0.71)	1.01	0.25	4.03	0.99
Race	0.26 (0.79)	1.29	0.28	6.03	0.75
Race by Group	3.06 (1.49)	21.23	1.15	390.74	0.04

Note: B = Unstandardized coefficient; SE = Standard Error; CI = Confidence interval; SES = socioeconomic status; LOC = Loss-of-Control-Eating.
